# Cimifugin suppresses allergic inflammation by reducing epithelial derived initiative key factors *via* regulating tight junctions

**DOI:** 10.1111/jcmm.13204

**Published:** 2017-06-09

**Authors:** Xiaoyu Wang, Xiaoyan Jiang, Xi Yu, Hailiang Liu, Yu Tao, Guorong Jiang, Min Hong

**Affiliations:** ^1^ Jiangsu Key Laboratory for Pharmacology and Safety Evaluation of Chinese Materia Medica Jiangsu Key Laboratory of Pediatric Respiratory Disease Nanjing University of Chinese Medicine Nanjing China; ^2^ Suzhou Hospital of Traditional Chinese Medicine Suzhou China

**Keywords:** cimifugin, atopic dermatitis, TSLP, IL‐33, tight junctions

## Abstract

Cimifugin is a bioactive component of *Saposhnikovia divaricata*, a Chinese herb for treating allergy. Our previous studies demonstrated that cimifugin inhibited allergic inflammation efficiently. This study aims to determine the mechanism of cimifugin on epithelial cells in allergic inflammation. Mice were sensitized and challenged with FITC to establish type 2 atopic dermatitis (AD) model. The initial stage of AD model, in which mice were just sensitized with FITC, was established *in vivo* and immortalized human epidermal (HaCaT) cells were utilized *in vitro*. Initiative key cytokines, TSLP and IL‐33, were measured by ELISA, the junctions in ECs were observed by electron microscopy and TJs (CLDN‐1, occludin and CLDND1) were assessed by Western blot, immunohistochemistry and immunofluorescence. The results showed that TSLP and IL‐33 were inhibited significantly by cimifugin in the initial stage of AD model. Simultaneously, cimifugin reduced the separated gap among the epithelial cells and increased the expression of TJs. Similar effects on TSLP/IL‐33 and TJs were obtained *in vitro*. The effect of cimifugin on TSLP decreased significantly when expression of CLDN1 was interfered with siRNA and this implied cimifugin inhibits initiative cytokines through restoring TJs. Furthermore, cimifugin administered only in the initial stage obviously attenuated the ultimate allergic inflammation, which indicate that impacts of cimifugin in the initial stage on TSLP/IL‐33 and TJs are sufficient for suppressing allergic inflammation. This study not only revealed the mechanisms of cimifugin, but also indicated the possibility of initiative key cytokines and TJs as therapeutic targets.

## Introduction

Cimifugin is an effective and main component of *Saposhnikovia divaricata* 1, 2. The dried roots of *S. divaricata*, called ‘Fang‐Feng’ in Chinese, are well known as a basic traditional Chinese medicine for thousands of years. ‘Fang‐Feng’ has anti‐inflammatory and anti‐allergy activities and is widely used for treating allergy, rheumatism, headache and convulsion, especially used as the first choice for allergic dermatitis and skin pruritus. The main components in ‘Fang‐Feng’ are cimifugin and prim‐o‐glucosylcimifugin as types of chromones. Prim‐o‐glucosylcimifugin is regarded as the quality standard for ‘Fang‐Feng’ and would transform into cimifugin *in vivo* 3, which have pharmacological activities with anti‐inflammation and detumescence 4. Previously, we reported cimifugin administered in the sensitization phase of mouse atopic dermatitis (AD) model significantly inhibited allergic inflammation 5. However, the mechanism is unknown and it's worth exploring the impact of cimifugin on allergic inflammation from the very beginning of the disease.

Allergic disease is the immune response caused by antigenic stimulation and most allergic diseases are mediated by Th2 lymphocytes. In clinic, corticosteroids, antihistamines, leukotriene modifiers, anticholinergics, β‐agonists and anti‐IgE preparations were used to treat allergic diseases. These drugs were effective at controlling the symptoms of allergy, however, reducing the relapse of allergy remains to be a worldwide problem 6. It is noteworthy that ‘Fang‐Feng’ appears features of reducing the relapse rate and recurring severity of allergy as a major component of well‐known Chinese prescription Yu‐ping‐feng‐san 2. Presumably, ‘Fang‐Feng’ may have distinctive action mechanism in allergic diseases. In traditional Chinese medicine, effects of ‘Fang‐Feng’ are described as ‘dispelling wind and relieving the exterior’. Thus we speculate that its mechanism of anti‐allergy might be related to its effect on epithelial cells (ECs), the very exterior of the body, in the skin.

Researchers have come to realize that ECs play a critical role in stimulating and regulating local immune responses 7. Studies of initiative key factors derived from ECs, thymic stromal lymphopoietin (TSLP) and interleukin (IL) ‐33, have provided important evidence that ECs can regulate the immune response to initiate the allergic response. TSLP is an IL‐7‐like cytokine that potently induces deregulation of Th2 responses 8, and IL‐33 is a cytokine of the IL‐1 cytokine family, as a key initiator of type 2 immunity found during allergic inflammation. TSLP and IL‐33 are hallmark features of allergic inflammatory diseases such as asthma and AD 9.

A critical role of ECs is forming a physical barrier protecting the body from inhaled harmful substances 7. The skin barrier is primarily determined by the integrity of intercellular junctions [i.e. tight junctions (TJs), adherent junctions and desmosomes], through which ECs are connected to each other, ultimately sealing off the paracellular space 10, 11. TJs form the most apical intercellular junction between ECs, providing functional polarity between the apical and basolateral domains 12, 13. TJs consist of different transmembrane proteins, including occludin 14, tricellulin and the claudin family 15.

Multiple disorders, such as asthma 16, inflammatory bowel disease 17, functional dyspepsia 18 and AD 19, have been linked to defective or altered TJs function. Barrier defects lead to ECs producing a set of prototypical cytokines (TSLP, IL‐33) that activate DCs to promote Th2 cell immunity 20, 21, 22. A loss of junctions in cultured ECs results in the increased production of TSLP 23, and this event could constitute an early step in the break of allergy milieu leading to Th2 cell sensitization and AD development.

Although there are plenty of evidence that ECs involved critically in allergic inflammation in recent researches, especially in the initiation of allergic response, it is hard to find the effects of existing anti‐allergic drugs on TSLP/IL‐33 or TJs expressed on ECs. We speculate that the effect of cimifugin maybe relate to its regulation of ECs based on the support of Chinese medicine theory and recent researches mentioned above. Specifically, we need to answer whether cimifugin could regulate initiative key factors, including TSLP and IL‐33, and their upstream regulators TJs derived from ECs and whether they could be adjustable targets in the treatment of AD.

## Materials and methods

### Materials

Cimifugin was purchased from National Institutes For Food And Drug Control (Shanghai, China, 20120511, Purity: ≥99%). Dexamethasone was a product from TianYao Pharmaceutical Co., Ltd (Hubei, China).

### Animals and cells

BALB/c mice were purchased from Shanghai Slac Laboratory Animal Company. All animals were maintained at Nanjing University of Chinese Medicine under specific pathogen‐free conditions at 18°C–25°C and 50–60% humidity, and were used at 6–10 weeks of age. All procedures involving animals were approved by the Animal Care and Use Committee of Nanjing University of Chinese Medicine and strictly performed according to the Guide for the Care and Use of Laboratory Animals. HaCaT cells (Immortalized human keratinocyte) were purchased from the Cell Bank of the Chinese Academy of Medical Sciences (Beijing, China) and cultured in MEM medium (Hyclone, Thermo scientific, Waltham, USA) supplemented with 10% foetal bovine serum (Capricron scientific, Ebsdorfergrund, Germany) at 37°C and 5% CO_2_.

### Initial stage of AD model *in vivo*

BALB/c mice were treated with 0.6% fluorescein isothiocyanate (FITC; Sigma, St. Louis, USA) in 20 μl acetone and dibutylphthalate (1:1, vehicle) on both ears on day 1 and 2, and killed on day 3. 20 μl acetone and dibutylphthalate was applied as vehicle control (control group). FITC treated mice were administered once daily with cimifugin (12.5 or 50 mg/kg, intragastrically), dexamethasone (DEX, 0.67 mg/kg, intraperitoneally) or normal saline as negative control (model group) 2 days before treatment with FITC until day 3 of the model. Both ears were removed and ground into homogenates with ice‐phosphate‐buffered saline (PBS), and the homogenates were centrifuged at 4000 × *g* at 4°C for 15 min. The concentrations of TSLP (both isoforms: lfTSLP and sfTSLP) and IL‐33 in ear homogenate were measured by ELISA kits (eBioscience, San Diego, CA, USA) according to the manufacturer's instructions. Total protein levels in the homogenates were examined by BCA kit (Thermo scientific). TSLP and IL‐33 protein levels were assessed with the formula: concentration of TSLP and IL‐33 in the homogenate/total protein (pg/mg).

### Cell culture and treatment *in vitro*

HaCaT cells were seeded into six‐well plates at a density of 1×10^6^ cells/ml and cells treated with or without cytokines incubated at 37°C under 5% CO_2_. Cells were pretreated with cimifugin or medium with 0.05% DMSO as vehicle (control) for 6 hr and stimulated with TNF‐α (20 ng/ml; R&D, Minnesota, USA) simultaneously for 12 hr. The concentrations of TSLP and IL‐33 in cell culture supernatant were quantified by Human TSLP ELISA kit (both isoforms: lfTSLP and sfTSLP, eBioscience) and Human IL‐33 ELISA kit (Becton, Dickinson and Company, USA). For immunofluorescence assay, cells were cultured and treated under the same condition as above, except they were seeded on the coverslips in 12‐well plates.

HaCaT cells were cultured and treated under the same condition as above. Cells were treated with recombinant human TSLP (lfTSLP, 50 ng/ml; Bioworld, China) or TNF‐α (20 ng/ml) up to 24 hr. The expressions of CLDND1, occludin and CLDN‐1 were detected by Western blot.

### Transfection with CLDN‐1 siRNA

HaCaT cells were seeded into 24‐well plates at a density of 1×10^5 ^cells/ml and incubated at 37°C under 5% CO_2_. Transfection with CLDN‐1 siRNA (Transheep, Shanghai, China) was performed using Lipofectamine 2000 (Life Technologies, Carlsbad, USA) according to the manufacturer's instructions. The siRNA‐lipid complexes were added to the HaCaT cells, and the medium was replaced 6 hr later. After transfected for 48 hr, the cells were treated with cimifugin or medium with 0.05% DMSO as vehicle (control) for 6 hr and stimulated with TNF‐α simultaneously for another 12 hr. The concentrations of TSLP in cell culture supernatant were quantified by human TSLP ELISA kit. To confirm whether the cells had been successfully transfected, the fluorescence microscope was used to confirm that the fluorescent signal of the siRNA was present in the cells. The sequence of the siRNA was CGAAAATGGACATTGAGAT.

### Quantitative real‐time PCR (qPCR) assay for mRNA expression of TSLP and IL‐33

Ear tissues of each group were homogenized in 1 ml of TRIzol (Life Technologies) using a glass homogenizer. The total RNA was isolated according to the manufacturer's protocol. The SYBR green PCR Master Mix (Thermo scientific) was used for real‐time PCR analysis. All reactions were run on an ABI 7500 Fast Real‐Time PCR System (Applied Biosystems, USA). The oligonucleotide sequences of the PCR primers (Sangon Biotech, Shanghai, China) were 5 ‐TACTATACTCTCAATCCTATCCCTG‐3 (sense, S) and 5 ‐ACTTCTTGTGCCATTTCCTG‐3 (antisense, AS) for TSLP (lfTSLP); 5 ‐TCCAACTCCAAGATTTCCCCG‐3 (S) and 5 ‐CATGCAGTAGACATGGCAGAA‐3 (AS) for IL‐33; and 5 ‐GGTTGTCTCCTGCGACTTCA‐3 (S) and 5 ‐TGGTCCAGGGTTTCTTACTCC‐3 (AS) for glyceraldehyde‐3‐phosphate dehydrogenase (GAPDH). The cycle time value of the interested gene was normalized with GAPDH of the same sample; fold induction of gene expression was calculated using the ΔΔCt method. Results obtained from each PCR were pooled and statistically analysed.

### Immunofluorescence assay

TSLP, IL‐33 and epithelial TJs proteins expressed in HaCaT cell were evaluated by immunofluorescence assay. The cells on the coverslips were fixed in methanol for 20 min. at −20°C and washed with PBS. For cytokines detection, the cells were permeabilizing with Triton X‐100 (Genview, Scientific Inc, Florida, USA) for 10 min., whereas this procedure was not needed for TJs proteins. The endogenous peroxidase was blocked by incubating in 3% H_2_O_2_ for 20 min., and non‐specific binding sites were blocked with 10% bovine serum albumin (BSA) for 1 hr at 37°C. The cells were probed with rabbit monoclonal antibodies against TSLP and IL‐33 (1:1000 dilution; Santa Cruz Biotechnology, Dallas, USA) or CLDN1 and occludin (1:1000 dilution; Abcam, Cambridge, England) at 4°C overnight. After repeated washes with PBS, the cells were probed with goat anti‐rabbit IgG conjugated to FITC (1:200; dilution; Santa Cruz Biotechnology) and 4′, 6‐diamidino‐2‐phenylindole (DAPI, Bioword, China) at a concentration of 0.1 μg/ml for 10 min. The labelled sections were observed with fluorescence optical microscopy (Mantra, PerkinElmer, Waltham, USA).

### Western blot assay for expression of TJs

Ear tissues of mice were removed and ground into homogenates with 20 μl/mg protein lysis solution (RIPA, phenylmethylsulfonyl fluoride and phosphatase inhibitor; 100:1:1). As for *in vitro* experiment, HaCaT cells were scraped from six‐well plates containing 100 μl of RIPA: phenylmethylsulfonyl fluoride (100:1). The samples (either from ear tissues or cells) were collected in microcentrifuge tubes, and lysed for 20 min. Then the homogenates were centrifuged at 13,201 g at 4°C for 10 min. The protein concentrations of the tissue or cell samples were determined using a BCA protein assay kit (Thermo scientific). Total protein extracts were resolved by 20% SDS–PAGE and transferred onto polyvinylidene difluoride membranes (Millipore, Darmstadt, Germany). After blocking with skim milk, the membranes were washed five times for 5 min. with Tris‐buffered saline, containing 0.1% Tween‐20 (TBST) at room temperature and then incubated with antibodies against CLDN‐1, CLDND1 or occludin (1:1000 dilution; Abcam) at 4°C overnight. After washing, membranes were incubated at room temperature with secondary peroxidase‐linked goat anti‐rabbit IgG (1:1000 dilution; Santa Cruz Biotechnology) for 2 hr. After washing, protein bands were detected by enhanced chemiluminescence (ECL kit; Millipore) and the protein expressions were quantified by ChemiScope analysis.

### Electron microscopy

The ear tissue specimen was first fixed with 2.5% glutaraldehyde in PBS for more than 4 hr; washed three times in PBS, then postfixed with 1% OsO_4_ for 1 hr and washed four times in PBS. The specimen was dehydrated by a graded series of ethanol (30%, 50%, 70%, 80%, 90% and 100%) for about 15 min. at each step and transferred to absolute acetone for 20 min. Afterwards, the specimen was placed in 1:1 mixture of absolute acetone and the resin for 1 hr at room temperature, then transferred to 1:3 mixture of absolute acetone and the resin for 3 hr and to final resin for overnight. After that, specimen was placed in capsules contained embedding medium and heated at 70°C for 48 hr. The 70 nm of specimen sections were stained by acetate and alkaline lead citrate for 15 min. respectively and observed in transmission electron microscope (JEOL, Tokyo, Japan).

### Immunohistochemistry for expression of TJs

CLDN‐1 and occludin in ear tissue samples were evaluated by immunohistochemistry (IHC). Antibodies against CLDN‐1 and occludin (1:1000 dilution; Abcam) were used for IHC. Dry tissue sections of 6 μm thickness at 60°C constant temperature box bake for 20 min. Slides were undergone dewaxing and hydration with sequential dimethylbenzene washes of 20 min. for twice, 100% ethanol washes of 10 min. for twice, sequential ethanol washes of 5 min. each starting 95% ethanol, followed by 80% and finishing with a 75% ethanol wash. Wash slides with PBS for twice, 5 min. each. Antigen was retrieved by citric acid buffer water bath heating at 95°C for 20 min., and then restored at room temperature. Wash slides with PBS for three times. Block endogenous peroxidase by incubating 20 min. in 3% H_2_O_2_ and wash slides with PBS for three times. Block non‐specific binding sites with 5% BSA for 20 min. The sections were probed with rabbit monoclonal antibodies against CLDN‐1 or occludin (1:1000 dilution; Abcam) at 4°C overnight. After repeated washes with PBS, the cells were probed with biotinylated secondary antibody (Zhongshanjinqiao; Beijing, China) for 20 min., and reveal the resulting peroxidase activity by incubating the slides with DAB for 7 min. Wash slides with PBS for three times. Counterstain for 1 min. with haematoxylin. Dehydrate slides with sequential ethanol washes of 5 min. each starting with 75%, followed by 80%, 95% and 100% ethanol wash, finishing with a dimethylbenzene washes. Seal slides and analyse by optical microscopy (Axion A1, Carl Zeiss AG, Germany). The mean DAB intensity was quantified by Mantra Quantitative Pathology Workstation (Mantra, PerkinElmer).

### Experimental FITC‐induced type 2 mouse AD model *in vivo*

BALB/c mice were treated with 1.5% FITC solution (in 20 μl acetone and dibutylphthalate (1:1, vehicle)) on the abdominal skin on days 1 and 2. On day 6, the right ears were elicited by 0.6% FITC solution. Acetone and dibutylphthalate was applied as vehicle control (control group). Mice were treated once daily with cimifugin (3.125, 12.5 or 50 mg/kg, intragastrically), DEX (0.67 mg/kg, intraperitoneally) or normal saline (model group) 2 days before sensitization until day 3 of the model (only administered in the initial stage of AD model). Ear thickness was measured 24 hr after elicitation on day 7 by thickness gauge (Mitutoyo, Japan) and changes in ear thickness were calculated. Histopathological changes of the ears were examined by haematoxylin and eosin (H&E) staining. The concentrations of IL‐4, IL‐5, IL‐9 and IL‐13 in ear homogenate were measured by ELISA kits (eBioscience) according to the manufacturer's instructions. Total protein levels in the homogenates were examined by BCA kit (Thermo scientific). Cytokines protein levels were assessed with the formula: concentration of cytokines in the homogenate/total protein (pg/mg).

### Statistical analysis

The data were expressed as means ± SD. Multiple groups’ comparisons were analysed by one‐way analysis of variance, and Dunnett's test was used for comparison between two groups, with GraphPad Prism 5 (GraphPad Software, San Diego, CA, USA). Statistical significance was set at *P* < 0.05.

## Results

### Cimifugin inhibited TSLP and IL‐33 production in the initial stage of AD model *in vivo*

**Figure 1:**
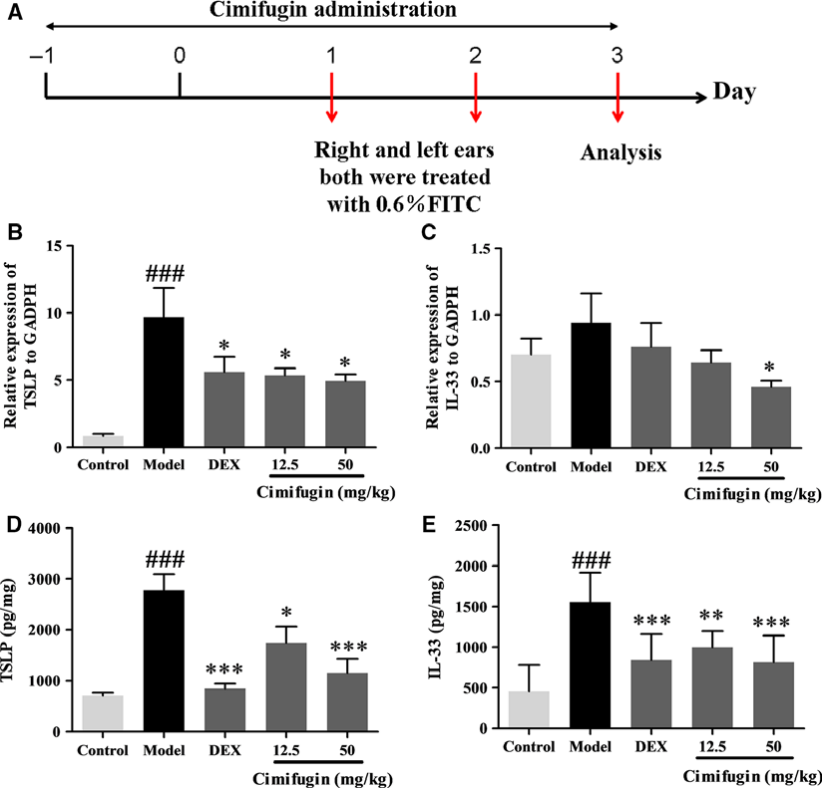
Effects of cimifugin on TSLP and IL‐33 in the initial stage of AD model *in vivo*. (**A**), Flow charts of the initial stage of AD model. (**B**,** C**), TSLP and IL‐33 mRNA expressions in ear homogenates were analysed by qPCR. (**D**,** E**), TSLP and IL‐33 protein expressions were analysed by ELISA and total protein were examined by BCA kit. TSLP and IL‐33 level were performed as concentration of TSLP or IL‐33/total protein (pg/mg). (mean + SD,* n *= 8, ^###^ *P *< 0.001 *versus* control,*P < 0.05, **P < 0.01, ***P < 0.001 *versus* model). The data are representatives of three independent experiments.

The initial stage of the mice AD model was established to observe the effect of cimifugin on TSLP and IL‐33 *in vivo*. Mice were treated once daily with 12.5 or 50 mg/kg cimifugin intragastrically, or 0.67 mg/kg dexamethasone (Dex) intraperitoneally, 2 days before treatment with FITC until day 3 of the model (Fig. 1A). We examined the changes in TSLP and IL‐33 mRNA and protein levels in ear tissue homogenates. Cimifugin, to varying degrees, reduced TSLP and IL‐33 mRNA compared with their levels in the untreated model group (Fig. 1B, C). Similar changes in protein levels were observed (Fig. 1D, E).

### Cimifugin reduced TSLP and IL‐33 production in HaCaT cells *in vitro*

**Figure 2:**
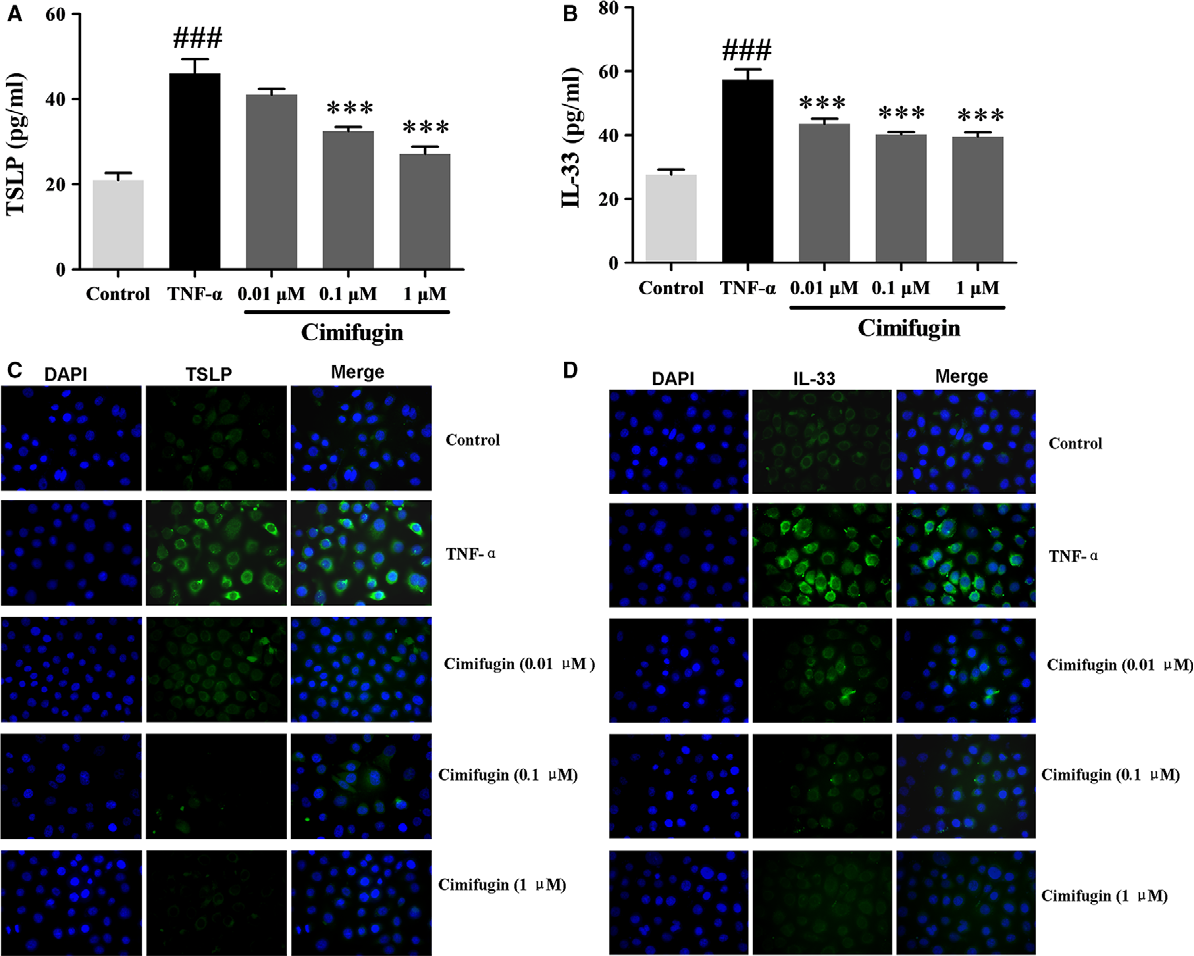
Effects of cimifugin on TSLP and IL‐33 in HaCaT cells *in vitro*. (**A**,** B**), Productions of TSLP and IL‐33 in HaCaT cells were analysed by ELISA. (**C**,** D**), TSLP and IL‐33 expressions were detected by immunofluorescence. (mean + SD,* n *= 3, magnification: ×100, ^###^ *P *< 0.001 *versus* control,***P < 0.001 *versus* TNF‐α). The data are representatives of three independent experiments.

To confirm the effects of cimifugin on the expression of TSLP and IL‐33, we examined the effects of cimifugin on the TNF‐α‐induced production of TSLP and IL‐33 in HaCaT cells *in vitro*. HaCaT cells were pretreated with cimifugin 0.01 μM, 0.1 μM, 1 μM for 6 hr, and stimulated with 20 ng/ml TNF‐α simultaneously for 12 hr. TNF‐α significantly increased the levels of TSLP and IL‐33 proteins, which were effectively down‐regulated by cimifugin (Fig. 2A, B). The immunofluorescence assay showed similar trends (Fig. 2 C, D). These results were consistent with previous observations *in vivo*.

### Cimifugin alleviated the junctions’ deficiency between epithelial cells in the initial stage of AD model

**Figure 3:**
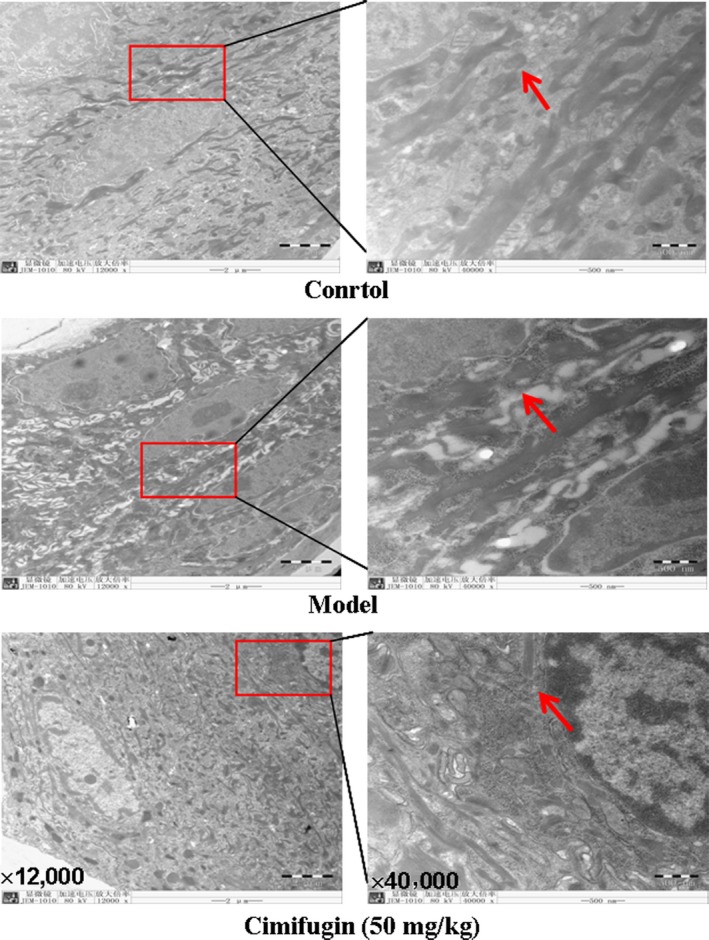
Effects of cimifugin on the ear skin epithelium in the initial stage of AD model. An electron microscopy observation has been performed on ears tissues of each group (*n *= 3, magnification: ×12,000; ×40,000). Arrowheads indicate the junction gap of epithelium. The data are representatives of three independent experiments.

Disturbed TJs may lead to increased susceptibility of epithelial cells to allergens, and then induced TSLP and IL‐33 production. We investigated the effect of cimifugin on epithelial junctions in the initial stage of the AD model with an electron microscopy. More separated gap and loose connection among the epithelial cells in the model mice were observed. Cimifugin alleviated pathologic status by reduced the intercellular gap and increased the epithelial connections in the initial stage of the AD model (Fig. 3).

### Cimifugin restored expression of epithelial TJs in the initial stage of AD model

**Figure 4:**
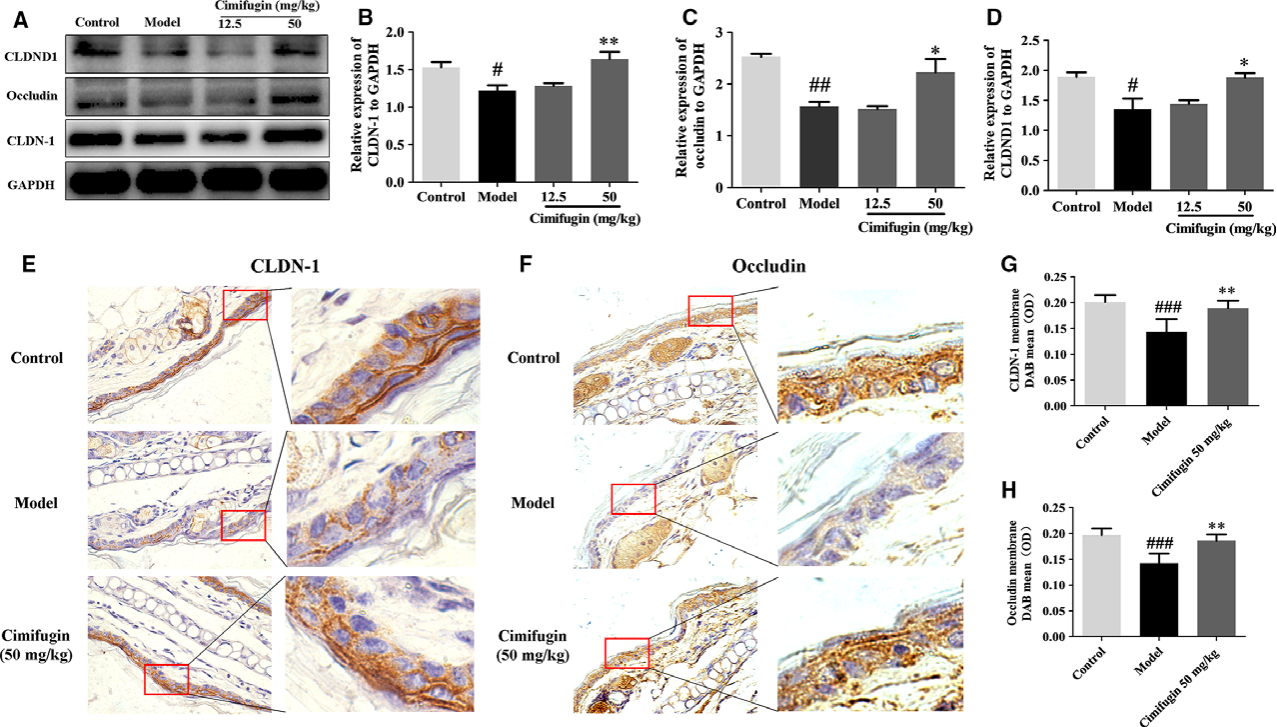
Effects of cimifugin on the TJs expressions in the initial stage of AD model. (**A**), CLDND1, CLDN‐1 and occludin expressions were analysed by Western blot (*n *= 3). (**B–D**), CLDND1, CLDN‐1 and occludin expressions relative to GAPDH were quantified by ChemiScope analysis. (**E**), Immunohistochemical analysis of CLDN‐1 and occludin expression (*n *= 5, magnification: ×630). (**F–H**), CLDN‐1 and occludin mean DAB intensity were quantified by Mantra Quantitative Pathology Workstation (mean + SD,* n *= 5, ^#^ *P *< 0.05, ^##^ *P *< 0.01, ^###^ *P *< 0.001 *versus* control, *P < 0.05, **P < 0.01 *versus* model). The data are representatives of three independent experiments.

The effects of cimifugin on epithelial tight junction proteins of ear skin epithelial cells on the initial stage of AD model were investigated by Western blot and immunohistochemistry. Impaired protein levels of CLDND1, CLDN‐1 and occludin were detected in the model compared with that in control group. Nevertheless, obviously increased CLDND1, CLDN‐1 and occludin proteins were found in cimifugin treated group (Fig. 4A–D). In addition, immunohistochemistry staining found that a relatively lower expression of CLDN‐1 and occludin in biopsy specimens of the model mice and cimifugin significantly improved the expressions of CLDN‐1 and occludin, in quantification of mean CLDN‐1 and occludin DAB intensity (Fig. 4E–H). These results indicated that epithelial TJs proteins could be restored by cimifugin in the initial stage of AD model.

### Epithelial TJs were regulated by cimifugin *in vitro*

**Figure 5:**
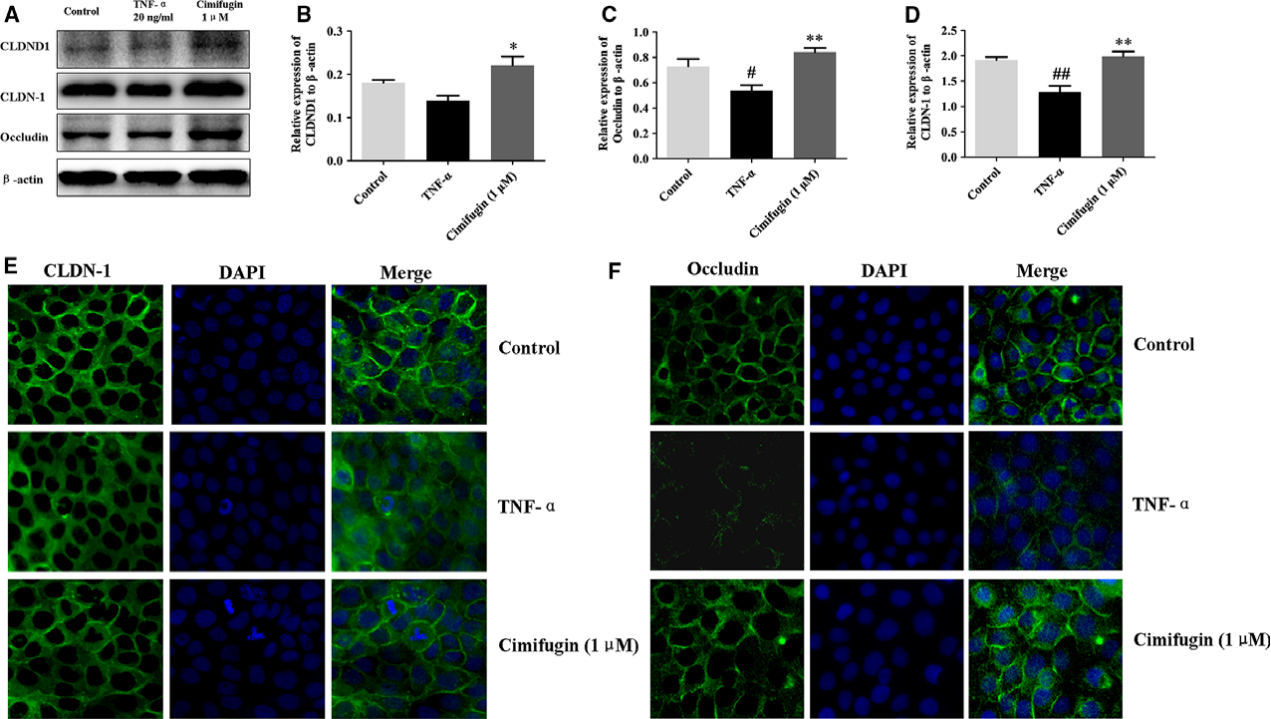
Effects of cimifugin on CLDND1, CLDN‐1 and occludin in HaCaT cells. (**A**), CLDND1, CLDN‐1 and occludin expressions were analysed by Western blot in HaCaT cells (*n *= 3). (**B–D**), CLDND1, CLDN‐1 and occludin expressions relative to β‐actin were quantified by ChemiScope analysis. (**E**,** F**), immunofluorescence quantification of CLDN‐1 and occludin expressions (*n *= 3, magnification: ×200; mean + SD,* n *= 3, ^#^ *P *< 0.05, ^##^ *P *< 0.01, *versus* control, *P < 0.05, **P < 0.01 *versus* TNF‐α). The data are representatives of three independent experiments.

HaCaT cells were treated with cimifugin 1 μM for 6 hr, and 20 ng/ml TNF‐α simultaneously for 12 hr. CLDND16, CLDND1, CLDN‐1 and occludin were detected by Western blot and immunofluorescence. A marked reduction in CLDND1, CLDN‐1 and occludin was detected by the Western blot in HaCaT cells treated with TNF‐α, whereas cimifugin significantly elevated CLDND1, CLDN‐1 and occludin expressions (Fig. 5A–D). Immunofluorescence staining showed an obvious weak arrangement of CLDN‐1 and occludin in the presence of TNF‐α. As a contrast, cells treated with cimifugin showed a trend towards higher expression of CLDN‐1 and occludin (Fig. 5E, F). These results were consistent with previous observations *in vivo* and implied that regulation of epithelial TJs might be an important mechanism of cimifugin.

### Cimifugin inhibited epithelial derived initiative key factor *via* regulating TJs

**Figure 6:**
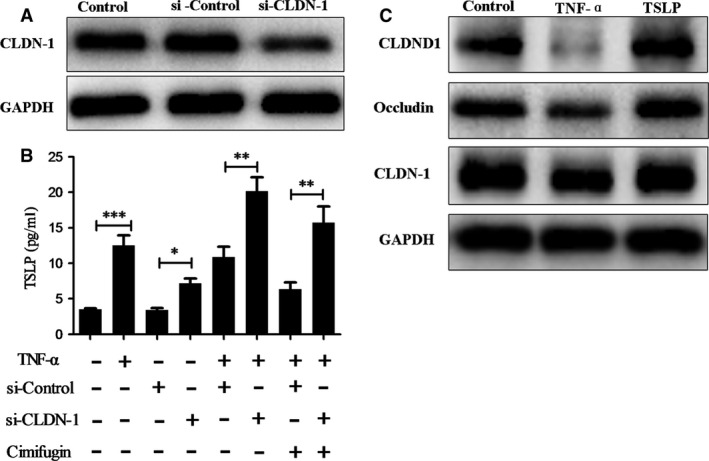
Cimifugin inhibited TSLP expression *via* regulating TJs. (**A**), Transfecting effect of CLDN‐1 siRNA was analysed by Western blot in HaCaT cells. (**B**), Productions of TSLP in HaCaT cells were analysed by ELISA. (**C**), CLDN‐1 expression was analysed by Western blot. (mean + SD,* n *= 3, *P < 0.05, **P < 0.01, ***P < 0.001). The data are representatives of three independent experiments.

To investigate whether cimifugin affected TJs first and then inhibited cytokines, the CLDN‐1 siRNA was transfected into HaCaT cells. When cells were transfected with the CLDN‐1 siRNA, CLDN‐1 expression was greatly reduced (Fig. 6A) and TSLP increased significantly when cells were transfected with CLDN‐1 siRNA. However, the inhibitory effects of cimifugin on TSLP failed to reach the previous level when interfered with the expression of CLDN1 (Fig. 6B). Whereas considering the effect of TSLP on TJs, the HaCaT cells were treated with 50 ng/ml recombinant human TSLP. No significant effect was detected of TSLP on the expression of TJs which implied cimifugin could not affect TJs through inhibiting TSLP (Fig. 6C). Together, these results indicated that the inhibitory effects of cimifugin on TSLP was CLDN‐1 dependent, that is cimifugin reduce initiative key factor *via* regulating TJs.

### Allergic inflammation was attenuated by cimifugin administered *only* in the initial stage of AD model

**Figure 7:**
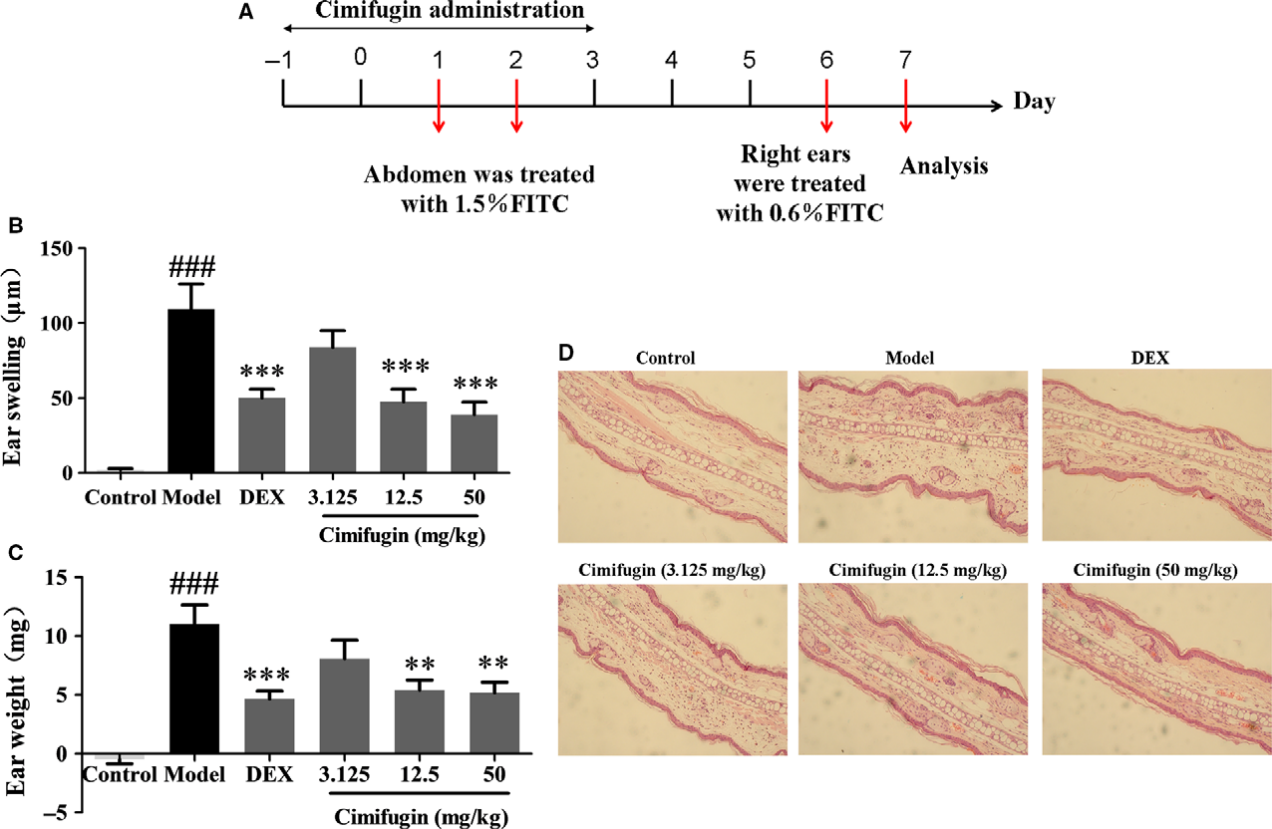
Effects of cimifugin administered only in the initial stage on ear thickness and histopathological changes in AD model. (**A**), Flow charts of cimifugin administration in AD murine model. (**B**,** C**), Effect of cimifugin on ear thickness and ear weight (mean + SD,* n *= 8, ^###^ *P *< 0.001 *versus* control, **P < 0.01, ***P < 0.001 *versus* model). (**D**), Histopathological changes examined by H&E staining. The data are representatives of three independent experiments.

**Figure 8:**
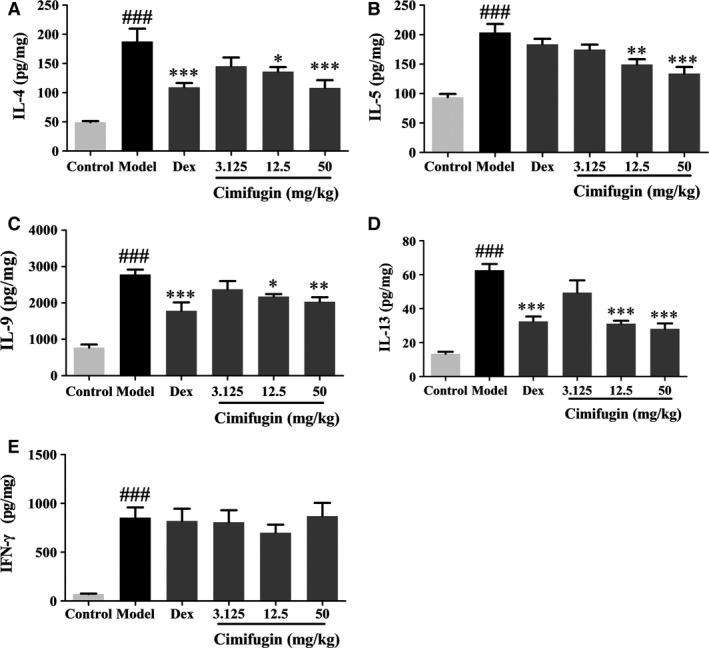
Effects of cimifugin administered only in the initial stage on Th2 cytokines in AD mice. (**A–D**) IL‐4, IL‐5, IL‐9 and IL‐13 expressions were detected by ELISA. (**E**) Th1 cytokine IFN‐γ expression was detected by ELISA. (mean + SD,* n *= 8, ^###^ *P *< 0.001 *versus* control, *P < 0.05, **P < 0.01, ***P < 0.001 *versus* model). The data are representatives of three independent experiments.

To determine whether the effects of cimifugin on TSLP/IL‐33 and TJs in the initial stage of ACD could ultimately influence the outcome of allergic inflammation, cimifugin was administered only in the initial stage. Mice were treated once daily with cimifugin (3.125, 12.5 or 50 mg/kg, intragastrically), DEX (0.67 mg/kg, intraperitoneally) or normal saline (model group) 2 days before sensitization until day 3 of the model (only administered at initial stage of AD model) (Fig. 7A). Allergic inflammation was determined on day 7. By measuring ear swelling and ear weight, we demonstrated the marked alleviation of inflammation by cimifugin in mice with FITC‐induced AD (Fig. 7B, C). In terms of pathological changes, thickening of the epidermis and infiltration of inflammatory cells were evident in AD mice compared with that in control mice. With the treatment of cimifugin, the thickening of epidermis and infiltration of inflammatory cells were effectively alleviated (Fig. 7D). The levels of Th2 cytokines (IL‐4, IL‐5, IL‐9, IL‐13) were significantly higher in the AD model, whereas decreased in cimifugin treated mice. No significant impact of cimifugin on Th1 inflammatory cytokine IFN‐γ was observed in this model (Fig. 8). These results indicated that the effects of cimifugin in the initial stage of AD model (day 1 to day 3), including regulation on initiative key factors and TJs of epithelial cells, are sufficient to suppress the eventual allergic inflammation (day 7), in the mouse AD model.

## Discussion

AD is a chronic, recurrent and inflammatory skin disease, characterized by chronic inflammation, impairment of the cutaneous‐epidermal barrier and hypersensitivity to environmental allergens induced by immunoglobulin E (IgE). In recent years, more and more studies recognized the critical role of epithelial cells in allergic inflammation. Cimifugin is an effective component of ‘Fang‐Feng’ with an efficacy of dispelling wind and relieving the exterior. The Chinese medicine theory suggests that its mechanism of anti‐allergy might relate to its effect on ECs.

TSLP and IL‐33 derived from ECs are master switches of allergic inflammation 24, 25, 26, suggesting that it might be the most important target interfering with the initial stage of allergic diseases 27, 28, 29. Studies have shown that epithelial cells are the main source of TSLP and IL‐33 production 20, 30, 31. These results directly confirm the link between TSLP, IL‐33 and allergic inflammation. In this study, we reported that cimifugin significantly inhibited the expressions of TSLP and IL‐33 in the initial stage of AD model and the results were validated *in vitro*, which indicated that regulating TSLP and IL‐33 might be the underlying mechanism of cimifugin.

It is currently believed that damage to epithelium leading to the epidermal barrier dysfunction is the primary one, and immunological aspects are a secondary phenomenon, which, however, further promote and support the development of AD 32. AD patients often associate with TJs disruption 33, 34 and decreased skin barrier function results in an increased uptake of allergens, which activates the immune system and results in inflammation. Researchers showed that the tracer molecules of 5 kDa–40 kDa are stopped at the TJs in cultured normal human epidermal keratinocytes after dermal injection 35. Cldn‐1 knockout mice showed a leaky barrier to a 557‐Da tracer 36. These evidence indicated that the decrease in tight junction could enhance the penetration of low molecular compounds such as FITC (approximately 389.4‐Da). Meanwhile, the function of keratinocytes can be changed by the decrease in tight junction. Epithelial barrier defect leads to the release of TSLP and IL‐33^37^. For example, a loss of E‐cadherin in cultured ECs results in the increased production of TSLP 23, and the disturbed desmoglein (DSG1) expression induces the expression of TSLP in keratinocytes in skin 38. Therefore, restoring the epithelial barrier function might limit the entrance of allergens and inhibit the expression of pro‐allergy key promoters.

In this study, we reported that cimifugin reduced the separated gap among the epithelial cells and increased the expressions of CLDN‐1, occludin and CLDND1 in the initial stage of AD model. Meanwhile, cimifugin restored CLDN‐1, occludin and CLDND1 expression *in vitro*. Furthermore, when cells were transfected with CLDN‐1 siRNA, the expression of TSLP increased significantly, whereas the inhibitory effects of cimifugin on TSLP decreased significantly compared with siRNA control. However, no significant effect was detected of TSLP on the expression of TJs, this result is consistent with the report of the view that the innate type 2 cytokines TSLP, IL‐25, IL‐33 had no effect on airway barrier integrity 39. Therefore, this indicated that cimifugin inhibited initiative key factors through regulating TJs.

So, could the impacts of cimifugin administered in the initial stage of AD model on TJs and TSLP/IL‐33 affect the outcome of allergic inflammation eventually? To illuminate this question, we designed the experiments that cimifugin was administered only in the initial stage of AD model. We found that cimifugin administered only in the initial stage attenuated thickening of the epidermis, reduced infiltration of inflammatory cells and decreased Th2 cytokines IL‐4, IL‐5, IL‐9, IL‐13 in the ear of the AD model. These results indicate that effects of cimifugin in the initial stage (day 1 to day 3), including downregulation of TSLP/IL‐33 and restoring of TJs, could eventually suppress the allergic inflammation, which occurred 4 days later (day 7). Therefore, regulation of TSLP/IL‐33 and TJs might be the mechanism for effect of cimifugin on allergic inflammation.

In summary, cimifugin significantly attenuated allergic inflammation by reducing TSLP and IL‐33 production *via* regulating TJs. Given the beneficial for inhibition of allergic diseases, cimifugin could be used as a prophylactic and (or) therapy for patients with AD, even other types of allergy, hopefully. Furthermore, our data indicated that TJs and the initiative key factors derived from ECs could be regulated, which implied that ECs might be affected as target cells and TJs or initiative key factors might be target molecules in the clinical treatment of AD. A better understanding of the important role of TJs and initiative key factors would be crucial for the development of therapeutics targeting TJs or initiative key factors to treat AD and other allergic diseases.

## Conflicts of interest

The authors confirm that there are no conflicts of interest.
